# Case of Ascites during Pregnancy
*A very interesting case of ascites during pregnancy, in which the patient was repeatedly tapped, is to be found in this Journal for March, 1802. It is from the pen of Sir L. Maclean; and we hope to be able, in a future Number, to lay before our readers the valuable results of this gentleman's further experience on this subject.—Editors.


**Published:** 1824-10

**Authors:** H. L. Sanders

**Affiliations:** Surgeon.


### Art. VIII.—*Case of Ascites during Pregnancy. By H. L. Sanders, Esq. Surgeon.

The remarks of a contemporary Journalist (Dr. James Johnson) have elicited from me the following case, which, if you deem it worthy a place in your valuable Journal, you will oblige me by inserting.

Mrs. Flowers, a coachman's wife, and the mother of four children, of a phthisical habit, ætatis thirty-seven, was seized with peripneumonic symptoms, about the fourth month of her pregnancy. She was freely and frequently bled, and, with the employment of antiphlogistic expectorant remedies, under the superintendance of a neighbouring practitioner, was much relieved. About the fifth month, her difficulty of breathing returned: she was again copiously bled by the same practitioner. Soon after this her feet began to swell, and symptoms of ascites and hydrothorax were but too apparent. She sought the advice of the physician-accoucheur to a neighbouring Dispensary, who permitted me to take charge of her, seeing her occasionally with me.

A cough (of the dry kind), dyspnœa, scantiness and almost total suppression of urine, general tenderness over the abdominal integuments, rapid collection of fluid within the cavities, a sensation as though the fœtus were unusually low and weighty, with a mind pertinaciously rivetted on the idea that she should die undelivered, with the greatest horror of such an event, were among the most prominent of my patient's complaints: the respiration being distressingly laborious, and only tolerable in an upright position; urine thick and very scanty, bowels obstinately costive, and sleep very much disturbed.

These symptoms were occasionally alleviated, and the patient's mind soothed, by the exhibition of conium, digitalis, squills, salines, &c. with frequent and large doses of castor-oil, and continued without much intermission until the ninth month of her pregnancy, when periodical pains, with sanguineous discharge, and other symptoms of speedily approaching labour, came on. Labour, however, did not take place until another month had elapsed; during which time the ascitic symptoms were greatly aggravated; her cough and dyspnœa (which was

---

* A very interesting case of ascites during pregnancy, in which the patient was repeatedly tapped, is to be found in this Journal for March, 1802. It is from the pen of Sir L. Maclean; and we hope to be able, in a future Number, to lay before our readers the valuable results of this gentleman's further experience on this subject.—Editors.

partially relieved by blistering,) was distressing; her mental agitation was so great as to simulate mania. On that day four weeks on which her symptoms began to increase, she was delivered, after a natural and easy labour of about ten hours' duration. The infant, a boy, (the most emaciated I had ever seen,) died about ten days after in a convulsive paroxysm. On the third day, milk appeared in both breasts, but she could not be prevailed upon to suffer the infant to suck, as placing it to the breast produced the most unconquerable delirium. On the death of the child, her milk speedily receded. During the recumbent posture, her ascitic symptoms were much ameliorated; her mind became tranquil, and slight hopes were entertained by her friends of her recovery. On resuming her domestic avocations, her phthisical symptoms returned, and after lingering four months longer she died.

*Remark.*—I conceive the hydropic tendency was much increased by the frequent and copious bleedings during the incipient stages of pregnancy, and that the inflammatory appearance of the blood, usual during that state, might have urged its abstraction to a larger extent than, under other circumstances, might have appeared warrantable. When ascites became evident, although the symptoms were greatly aggravated by pregnancy, I conceive that paracentesis abdominis was perfectly inadmissible, by reason of the morbid condition of the constitution generally. This patient had an idea that her period of gestation exceeded the usual term by four weeks; and, from the very minute inquiries that I instituted, I am decidedly disposed to support the same opinion, when I reflect on the naturally feeble and artificially reduced powers of the constitution of the patient, insufficient either to complete the development of the embryo, or to enable the uterus to perform its last office—the expulsion of the fœtus.

*Cleveland-court, St. James's-place; Sept. 15th, 1824.*

